# **Voices of a generation** the communicative power of youth activism

**DOI:** 10.1007/s10584-021-03211-z

**Published:** 2021-11-02

**Authors:** Elisabeth Eide, Risto Kunelius

**Affiliations:** 1 Department of Journalism and Communication Studies at OsloMet – Oslo Metropolitan University, Oslo, Norway; 2 Department of Media and Communication Studies, University of Helsinki, Helsinki, Finland

**Keywords:** Climate change, Fridays for future, Climate science, Youth activism, Climate communication

## Abstract

Drawing from interviews with 31 young leading climate activists from 23 countries across the world this article aims to capture the contribution of the recent youth climate movement to communicating climate science and politics. We show that from the point of view of the youth activists, the movement powerfully connects personal and local experiences and emotions with climate science. This has enabled the activists to construct an authentic, generational and temporal identity that has helped them to carve out an autonomous position and voice with considerable moral authority among existing climate policy actors. Claiming to represent the future generation, we conclude that activists have offered an important added value to climate science as new ambassadors for scientific consensus and climate mitigation. The youth movement and the added value it brings communicating climate science is an example of the dynamics of the formation of “relational publics” and emphasizes the need to understand better the networked communication landscape where climate politics is debated.

**Voices of a generation.**

The communicative power of youth activism.

*But that is easily fixed – just start to listen to the rock-solid science instead. Because if everyone listened to the scientists and the facts that I constantly refer to, then no one would have to listen to me or any of the other hundreds of thousands of schoolchildren on strike for the climate across the world.*

(Greta Thunberg, 2019: 32–33).

## Introduction

Born in 2003, or “at 375 ppm” as her Twitter handles declares, Greta Thunberg was first observed publicly as a lone 15-year-old demonstrator in front the Swedish parliament in the fall of 2018. She then rose remarkably quickly to a spearhead of a global youth climate movement and an influencer with 5 million followers on Twitter. Despite the unique celebrity status of Thunberg, *Fridays for Future* is a diverse grassroots movement, which mobilizes masses of young people to stay away from school – and thus symbolically question the plausibility of the future that education is supposed to be preparing them for. This captured global public attention and allowed the movement to add a new accent to the contentious and often divisive debate about the climate crisis.

Climate science has played a central part in the rhetoric of the youth movement throughout its existence. The dire message of climate science has served as the touchstone for the activists. At the same time, activists have shaped a new phase of climate debate with its increasingly critical and urgent demands for action. In this article we argue the movement serves as an example of building of significant “communicative power” (Arendt, 1970). Enabled by the current global, networked communication infrastructure the movement has crafted a relatively autonomous identity in the global debate and has re-energized the message of climate science and expertise with a new distinct voice as well as a kind of moral authority and resonance. Understanding how this space in the contested field of climate politics was carved demands that we explore the youth movement leaders as emerging actors in specific network of other actors in the global climate politics. This approach relies on conceptualizing a “relational public” essentially as a network of not only flows of communication but of “shared stories and collective concerns” that can reinforce of transform identities, movements, and systems of power” (Starr, 2021). Understanding the network and interplay of the youth movement, climate science, and other actors of climate politics may highlight parts of the dynamic of the current communication environment in which scientific expertise about the global climate crisis plays out.

In this article, we elaborate this network by drawing from the youth activists’ own experiences. Analysing in-depth interviews with young leaders from 23 countries around the world, we aim to identify the “added value” that the youth movement has brought into global climate communication. We trace this contribution by answers to three interrelated questions.

RQ 1: How did the activists express their relationship to climate science?

RQ2: How did the activists rely on their own lived, local, and emotional experiences in articulating and mobilizing the movement?

RQ 3: How did the activists situate themselves in the contemporary landscape of climate communication and politics, and what is their added value to ongoing climate discourses?

Each of these questions points to distinct fields of earlier inquiry. We will therefore begin (section 2) by introducing (through selected earlier scholarship) three conceptual threads that have informed our analysis: 1) challenges of climate science communication, 2) the specific nature of the youth movement itself, and 3) the broader communication landscape (both in terms of media infrastructure and political division). We will then present the empirical material and discuss our methodological approach in section 3. This is followed by the empirical analysis focusing on the three research questions (sections 4, 5, and 6). While these sections provide distinct findings and reflections on these specific themes, our main argument is a synthetic one: if we are to understand the specific value of the youth movement – and its lessons to climate science communication – it is crucial to see how the knowledge *authority* drawn from climate science, the *authenticity* drawn from personal and lived experience, and the relative *autonomy* of the movement in relation to other actors were combined and mutually constructed. The concluding section, then, presents the key findings as interrelated elements that shape the communicative power of the movement.

## Contexts: Science communication, youth activism and media landscapes

### Science communication

The IPCC 1.5 C report in October 2018 created a crucial factual backcloth for the demands of the youth movement and played a key role in bringing about a new rise in global media attention to climate change (Boykoff et al., 2021). Initiated after the Paris Agreement (COP21, 2015), it repeated the basic message of earlier IPCC reports and communicated that youth activists and their generation will inherit, along with between 8.4 billion and 11.3 billion others, a planet that will be 0.8–2.6 degrees warmer with sea levels 5–32 cm higher (IPCC 2013, synthesized in O’Brien et al., 2018). The report also underlined the incompatibility of current carbon emission paths with national pledges to the Paris Agreement (IPCC 2018; see also Aldy et al., 2016; Rogelj et al., 2016).

More importantly, however, the report provided a new frame of interpretation. It clarified, in relatively accessible and simple terms, the difference in consequences between 1.5 °C and 2 °C of average global warming (IPCC, SPM 2018). Thus, it underscored that while past emissions have already baked risky future changes into the climate system, the level of ambition in mitigation and other action can still make a difference. This adjusted framing effectively re-energized the climate debate by showing that even if the 2 °C political targets set by the Paris Agreement 2015 were to be reached, it would still not make things right. The message echoed the core point made by a coalition of the most vulnerable countries^1^ that played a very active role at the Paris COP21 by pressing for a more ambitious target to be included in the text of the final accord (e.g., Boykoff, 2019: 82–84).

The youth movement did not emerge as a direct consequence of the 1.5 °C framing. However, the IPCC, by emphasizing the need for “unprecedented” and “systemic” changes, provided key ingredients for it. During 2018 and 2019, scientific messaging, energized activism, and dramatic extreme weather events intersected in powerful ways. At this conjuncture, the overall discourse showed signs of change. Several actors, such as the European Parliament and some leading mainstream news organizations began to replace the word ‘change’ in ‘climate change’ with ‘crisis’ or ‘emergency’. The global *Fridays for Future* (FFF) movement contributed to this heightened attention and sense of political urgency. At the same time, the interplay between scientists, politicians and the media created favourable conditions for the movement to appear and spread.

This interplay underscores the complexity of the contemporary science communication, which is still often based on the idea of an information ‘deficit’ implying a one-way flow of information. Aiming at better ‘science literacy’ among lay people, this model assumes an ideal of wisdom flowing from scientists to their audiences. In contrast, science communication scholars have long stressed the need for alternative models. For instance, Secko, Amend and Friday (2013, p. 6) have pointed to the role of *context* (developing different messaging for different audiences), *lay expertise* (highlighting interaction between scientists and lay people) and *public participation* (democratizing science through more engaging communication).

Dialogic models, such as lay expertise and participatory science, however, do not offer simple solutions, particularly when we are faced with complex issues such as modelling of climate futures. The role of IPCC scientists in delivering descriptive and policy-relevant, but not policy prescriptive, knowledge, (IPCC, 2015), after all, begs for a considerable amount of authority, as it deals with communicating *probable* futures with a complex *risk-uncertainty* vocabulary. This dilemma has, of course, seen some scientists stretch the ‘policy-relevant’ statements to saying that ‘substantial and sustained reductions in greenhouse gas emissions’ are required (see e.g. author, 2017, xxx & author 2019), thus offering a chance for politicians and activists to translate scientific evidence more easily into calls for climate action.

Seeing science communication as a cooperative and networked effort may help scientists to retain their strengths while allowing other actors to re-formulate climate knowledge to a wider range of communicative genres and gain better resonance with different contexts and people (Boykoff, 2019). Modelling science communication as a co-productive action poses the question about the *specific added value* and contribution of the youth movement in communicating the climate information. This leads our attention to earlier research on climate activism.

### The nature of youth climate activism

Many scholars have focused on the *emotional* aspect of young climate activists and their expressed combination of fear, despair, and hope. A telling moment of this was Thunberg’s speech to the UN in the fall of 2019, stating: “I want you to panic.” (Thunberg, 2019). We should not look at such rhetoric merely as a communication strategy but also recognize the psychological burden that it expresses. In a situation where media has often stressed frames of ‘doom and gloom’ and disaster (O’Brien et al., 2018; Painter, 2017; O’Neill et al., 2015; O’Neill & Nicholson-Cole, 2009), fearfulness may indeed easily emerge.

Nairn (2019) has emphasized how different emotions may interfere with each other. In a study of youth in New Zealand, she found ‘burnout’ among activists who had previously experienced hope and positive aspirations that, in turn, led to their retreat from activism. On the other hand, the young people she studied seemed to generate hope by taking part in collective processes and engaging in climate activist groups, emphasizing the importance of networking (Nairn, 2019, 440; see also Kleres & Wettergren, 2017; Lee et al., 2020). As other researchers have observed, the “framing of climate change as an impending environmental disaster may contribute to a sense of despair and feeling of helplessness, which can lead to disillusion, apathy and inactivity” (O’Brien et al., 2018), while positive framings may generate a different kind of engagement and bring opportunities for action (ibid.). The movement has somewhat successfully presented itself through a synthesis of fear and hope as a necessary combination of emotions that climate politics needs.

The emphasis on emotions points to the way the youth movement strongly anchored to *personal experience* and its importance in politics. Fisher’s earlier interviews with international young climate activists highlighted the “transformative moments” in their lives “holistically recognizing the radical implications of climate change for both the environment and social justice may be the most effective for encouraging commitment” (Fisher: 2016, 242). Building on a long research track on youth and climate, Ojala, in turn, has shown how young people use “meaning-focused strategies” to evoke hope and cope with threats (Ojala, 2012). She has also highlighted the way in which such coping was linked to trust in other societal actors, the role of parents and the way they help youngsters to cultivate constructive reactions to negative emotions related to social problems (Ojala, 2019). While these findings partly resonate with our own, our respondents clearly emphasized peer-support in this emotional work and motivation for action (see also Wallis & Loy, 2021).

The overall *sociological profile* of youth climate activists has been highlighted in several recent studies, mostly focusing on the Global North (Wielk 2020; Nairn, 2019; O’Brien et al., 2018; Kleres & Wettergren 2017) although Fisher (2016) is a refreshing exception. De Moor et al. (2020) emphasized the large mobilization of a new age cohort (school pupils), especially girls; however, they also pointed towards a higher educational bias (see also Emilsson, Johansson & Wennerhag 2020). Scholars have also tried to capture the variations of political demands inside the movement. O’Brien et al. (2018) distinguished between *dutiful* (reformist)*, disruptive* (norm-challenging) and *dangerous* (mobilizing alternatives) dissent, arguing that these models of activism – and their creative combinations – will be needed for youth climate activism to have an impact. Other scholars also highlight how the radical potential of the movement might be overshadowed by the unifying perspective (Marguardt 2020; see also Ellisson et al. 2020; de Moor et al. 2020).

Overall, as Han and Ahn (2020) point out, the FFF movement has succeeded in mobilizing and incorporating multiple new voices into a joint movement. By doing so, youth activists have assumed a role as “environmental stewards” for the future, in contrast to some depictions of previous generations of young people, such as Millennials, as passive and self-centred.

### Communication landscape: Infrastructure and politics

Much transnational climate action has earlier occurred led by existing environmental movements; NGOs such as Greenpeace and Friends of the Earth, or umbrella NGOs explicitly concerned with climate change, such as Climate Action Network (Bomberg, 2012: 412). The general link between the power of such networks and digital communication has been discussed and theorized for more than a decade (e.g., Castells, 2009; see also Caren et al. 2020). However, in a relational analysis, two specific aspects of the broader communication environment of the youth movement deserve attention.

First, the youth movement emphasizes a particular potential of the contemporary communication infrastructure. The recent youth climate movement highlights an emotional, personal commitment and rhetoric, which has brought together activists from very different and diverse contexts. This echoes Bennett and Segerberg’s (2013) notion of “connective action”; the way in which the networked landscape of social media can cultivate *personal action frames* that allow people to join a “movement” for different reasons and confront “a situation that has to be changed” (ibid: 37). One distinct aspect of the current youth activists is their way of expressing themselves more consciously as inheritors of the planet. A parallel personal-generational argument is also articulated by grandparent action networks (see Lawson et al. 2018 on intergenerational learning). Furthermore, even if some of the local initiatives originate from existing organizations, the FFF and related actions have mostly built their own structures. The youth actions starting in 2018, in the organizational sense, may bear more similarities to separate ad-hoc initiatives, such as for example ‘Black Lives Matter’ (Lawson et al. 2018: 206).

Second, the rise of the FFF-movement has intersected with a tide of global, populist and often authoritarian, nationalistic political “backlash” (Inglehart & Norris, 2018), which has sharpened political polarization. As many observers have pointed out, this wave of populism (e.g., Moffitt, 2016) is, despite local nuances, a rising global characteristic of political cultures. The polarization of climate debates has been a key part of the global political opportunity structure that has shaped the movement. Aggressive reactions from one side of the climate debate may have built public acceptance and sympathy for the movement. At the same time, such alliances have created challenges to the whole political elite.

## Method and data: Listening to a diversity of voices

We look at climate change communication, science, and politics from the horizon of activists in the youth climate movement. We reflect on discussions with young climate activists from 23 countries around the world (Table 1). Altogether, 31 activists from all continents were interviewed by the members of the MediaClimate network^2^ during 2019 and 2020.

**Table 1:** Table 1

Country	Person(s)	Age at time of interview
Australia	Female	15
Bangladesh	Male	30
Brazil	Female	30
Canada	Female	20
Chile	Male Female Male	23 26 22
Denmark	Male	17
Finland	Male Female	17 23
France	Male	16
Germany	Male	16
India	Male	17
Indonesia	Male	25
Italy	Male	26
Japan	Male	18
Kenya	Male	30
Norway	Male Female	24 16
Pakistan	Male	26
Russia	Female	28
Slovenia	Male Female	22 18
South Africa	Female	18
Sweden	Female	19
Turkey	Female	16
Uganda	Female Male	22 28
U.S.	Female Male Female	17 18 12

### The sample/data

Youth climate activism varies across the world due to the different primary climate concerns, political contexts, and media environments. In this sense, the movement mirrors the general complexity of the global–local dimension of climate politics. The youth activism around the world also represents locally driven efforts, oftentimes with a loose and unofficial organizational structure. This means that finding a representative sample of activists across different parts of the world is challenging. Our 31 respondents speak not only from a variety of countries, but also from different roles within their local movements. Our material exposes a diversity of cultures, languages, and local conditions. Table 1 provides an overview of the interviews.

The young activists were between 12 and 30 years of age. Although we initially defined *young* people to be below 25, some (6 out of 31) interviewees are older.^3^ One of the three interviewees in the U.S. was only 12 at the time of interview, while all the others were 16 or older. Some were leaders of established NGOs with a history of being involved in climate and environmental activism. Other leaders had emerged through the FFF. Of the 31 interviewees, 14 were female and 17 were male.^4^ In most locations, the respondents clearly saw themselves as part of a global movement, but in some cases, they saw their own activism more as a related activity following its own logic. Some respondents came from extremely vulnerable developing countries, others mobilizing activism in countries featuring at the top of the UN Human Development Index. The interviews followed a semi-structured guide asking the respondents to explain and reflect on their:

1. personal inspirations to join the climate movement,
2. background and their motivation for action,
3. role and tasks in the local movement,
4. thoughts on what aspect of climate change were crucial locally,
5. experiences and practices of local organizing (events, decision-making, internal communication)
6. relationship to climate science and scientists,
7. relationship to established environmental NGO’s,
8. relationship to media and professional journalists,
9. their relationship to other political and social stakeholder (parties, politicians, teachers, police and others)

The interviews were conducted in several native languages, and our synthesis here relies on reporting, paraphrasing and translations of key quotations by members of the research network.

As this description suggests, the material is characterized by multi-dimensional diversity that stems from different roles of respondents, from varying contexts and cultures, different interviewers, and languages. While we strongly trust the validity and quality of the research network, we have approached the material cautiously and stress that the interpretations and conclusions—and the responsibility for them—are our own. In section 2, we introduced three interpretative frames that have guided our thematic, theoretically informed close reading of the material. Having done so, we want to acknowledge that we, of course, owe our voice to our colleagues (see Annex 1) and the respondents. Without their local knowledge and commitment, we would not have anything to say. In the end, findings and reflections come from an effort to *think with* a community of interested participants who helped us to look at youth climate action through the eyes of individuals involved in the movement.

## Youth movement and climate science

### Trust in science

The overwhelming starting point of our respondents was that science—and, by implication, the IPCC—should be taken seriously. Not surprisingly, almost all the youth activists took on the role of speaking in the name of science. Most interviewees considered Greta Thunberg and her steady dedication to climate science as a model of inspiration.

One interviewee from South Africa shared the same urgency as her Swedish colleague: “Because the way we’re going according to the IPCC and other credible sources, we are really starting to get to a point where if we don’t do anything quickly, we’re not going to be able to stop anything.”’ (South Africa, interview). A French activist said that he follows Valérie Masson-Delmotte,^5^ France’s internationally renowned climate scientist, on Twitter, in addition to Citoyens pour le Climat (citizens for climate), which communicates scientific discoveries to the public (France, interview). A Slovenian activist described herself as an ‘amateur meteorologist’ (Slovenia, interview).

Several respondents said they had read IPCC documents, such as the abbreviated summaries for policymakers (SPMs), while others had become aware of IPCC’s conclusions through social media and recommended scientific articles. Quite a few of the activists had been raised with this kind of scientific trust by environmentally conscious parents, among them well-to-do academics. This underlines the role of socialization in the cultivation of “meaning-focused coping” towards climate threats (Ojala, 2019) as motivating pro-environmental attitudes. However, it also points at the possibility of climate activism as an élite phenomenon that builds on generationally accumulated social and cultural capital. An Italian activist complained: “Scientific ignorance is widespread [here], much more than in other countries”. She felt that media “do not understand how *we are those in favour of science* while they [opponents] are against science and development.” (Italy, Interview). A Swedish activist had read parts of the full IPCC reports, concluding that, “when one reads them, one understands exactly how serious it is. I cannot understand how people can know of these facts and are (still) not willing to do anything” (Sweden, Interview). In turn, a Ugandan activist launched a critique, not of other people, but of global leaders for their “inability to take the necessary action”, explaining that, “scientists have provided the technical information” (Uganda, Interview). These activists’ trust in climate science was especially motivated by calls for action, since activism is a larger part of their social fabric. Thus, they provide a bridge from the climate scientists’ “policy relevant, but not policy prescriptive” status (IPCC mandate) to activities seeking concrete political remedies and changes.

### Science literacy and educators

Trusting in science does not mean that activists would not recognize the complexity of the climate science knowledge. A Danish activist expressed some scepticism towards “all these predictions of what will happen. Because with the climate crisis we [only] know how little we know” (Denmark, Interview). Nevertheless, while he demonstrated an awareness of the uncertainty aspects of climate science, he also declared that “you don’t really need all these statistics to explain the climate crisis. Because it is evident: […] we can’t continue doing things that harm the earth. And we know that.” (Ibid.) This points to a tacit understanding of how handling scientific certainty or consensus and public or political reasoning must, to a degree, follow different logics. It also shows that activists can—consciously and strategically—choose to take some distance from scientists. Communicating uncertainty is a particular challenge for climate scientists, who have seen uncertainties exaggerated in the media as indicators of “controversial science” (Painter, 2017).

A French activist blamed the media for giving too much space to “climate sceptics” and too little to scientists. He also criticized schools for the ignorance of his classmates, “who, for example, do not know what the IPCC is” (France, interview). A Slovenian activist, on the other hand, said that he had studied “a lot of environmental topics” at his primary school, also working practically to save the environment (Slovenia, interview). Acquiring knowledge through practice, and thus understanding the climate situation better, has been confirmed by a Norwegian study showing that the respondents who were engaged practically in climate or environment activities also trusted climate science significantly more than other respondents (Austgulen & Stø 2013). The Russian activist met people who asked how global warming could take place while they said,” it is only getting colder in Murmansk?” The activist also stressed her own learning curve: “Just a month ago I was totally clueless about this topic. But after researching the issue, I can explain [to these people] that weather and climate are not the same things.” (Russia, interview).

These excerpts show the importance of the larger social knowledge infrastructure in enabling active engagement with science. A Norwegian activist talked about how the IPCC’s 1.5 °C report of October 2018 played an important motivating role, and “particularly the SPM and the [translated] summary created by the Directorate of Environment. These documents were very concrete and received a lot of press coverage” (Norway, interview). The importance of local institutions, with their trust capital and ability to use local languages in disseminating climate science to broader segments of society is crucial. At the same time, however, this reveals important global inequalities: where local scientific and institutional structures are weaker, and where there are, for instance, no prominent local climate scientists or IPCC authors, translating and contextualizing global knowledge to local conditions is still a major obstacle (Nassanga et al. 2017; Nassanga & Rhaman, 2020).

Our interviewees raise the issue of how activism could also be seen as part of such a communication infrastructure of science. A young activist from Chile, for instance, registered a loss of biodiversity. ‘As a geographer I have studied the destruction of wetlands, these […] are incredibly useful for the climate […] but we are swapping them for sand and buildings’ (Chile, interview). Here is an example of how young people educate themselves with a purpose. Some interviewees, already in their 20s, had also undertaken the role of *educators*, realizing that many of their fellow citizens, need more climate knowledge.

The Indonesian activist had “educated the students ... not like a scientist; rather sharing or introducing these issues, because not all children—maybe in Indonesia, especially in Sumatra—have heard of the issue of climate change.” (Indonesia, interview). He had successfully combined lessons on climate change with the critical extinction of species, which his young students could observe around them. Likewise, the Pakistani activist conducted Climate Education camps, which became “the best experience so far in my activism”. He had gone to northern Pakistan, an area very prone to climate change, “and I was happy to see children full of zeal to learn about this issue” (Pakistan, interview). The Kenyan activist worked with other youths at universities and “we interact with school […] kids in terms of trying to nurture the culture of maintaining the environment from an early age” (Kenya, interview). Thus, the science is *passed on* by young volunteer teachers, experienced activists (who have interacted with scientists or are self-learned) to younger ones.

It is a paradox that while climate change “disproportionately affects those in the most disadvantaged groups”, the “climate engagement tends to increase with education and income” (Happer, 2019). For many, technology-based solutions that are recommended partly by scientists, partly by entrepreneurs and politicians, seem to be far beyond the imagination of disadvantaged groups: “investment in solar panels, electric cars, passive house technologies are unreachable” (Fox, 2019; see also Brisman, 2009). Additionally, features of climate activism such as changing consumer habits may be less attractive to the poor, writes North (2011). However, he concludes that unlike other global initiatives, “the range of spaces and scales at which climate activists organise is greater” (ibid.). The youth movement, then, can at its best play the role of “agents of climate science consensus” and occupy a particular educative place within societies with low climate literacy.

### Scientists and activists

Some respondents testified about interaction between scientists and the movement. One of the Norwegian activists said that academic institutions had unofficially sent their scientists to speak at activists’ rallies, and that scientists and authors had added their signatures to a protest published in *Aftenposten*, Norway’s largest subscription newspaper (Norway, interview).

A Canadian activist found herself at a European conference: “We had IPCC scientists, over 150 media present […] IPCC scientists, one of them, like broke down in tears and hugged me, saying: ‘you’re the reason I still wake up in the morning.’” Such a reaction from a renowned scientist points to the different roles played by the scientists and youth activists and shows how both can emotionally find a way to move together. The interplay between the experiences of citizens and scientists offers impressive openings for further learning, however. The same Canadian leader also told of other experiences of connecting with scientists: “I think you can call [a] sort of hotline of scientists, you know, like what’s this fact, and they help you” (Canada, interview). This special ‘reach-out’ from scientists to citizens may prove an interesting path for future communication.

Even if most climate scientists do not subscribe to activism, there are those who do (Ytterstad, 2014; author, 2017). In a series of interviews with leading climate scientists, some spoke about the temptation to be prescriptive. As one IPCC author expressed it: “[…] it is difficult not to have a more personal and subjective way of communicating. When you emphasise the dangers and the negative consequences, it should be okay to express hope that negotiations will lead to some positive results” (Author, 2017, 43). Another scientist said that reaching the 2-degree target must be linked to other societal problems, and underlined the complexity of the issue, which will “affect today’s youth during their entire life span”. The same scientist added that there might be a need for those who understand the science to dare “turn a little from *is* to *ought to*” (Author, 2017, 43, emphasis added).

## Lived experiences: The authenticity of the movement

Connecting global processes of climate change to local events has been a recurring issue among academics and journalists. Climate researchers are often careful when communicating about climate change processes linked to extreme weather events (Duarte, 2016; Duarte & Author, 2019), although they conclude with increased occurrences. IPCC special reports (IPCC 2012, 2014, 2018, 2021) suggest that some connections and practices of weather attribution serve the debate increasingly better (see also Painter & Hassol, 2020).

### Personal experiences

Our respondents’ personal experiences provide a powerful way of narrowing the gap between passive and active citizenship, global and local knowledge as well as anxiety and activism. This was well stated by an Australian activist. For her, everyday experience came first, followed by science and verification:

The thing that made me feel most connected to the issue was when people talk about their *experiences*, because it is inherently an issue that affects people […] so it was an initial response from the *personal experience* that climate change has an impact on people’s personal lives [that] led me to read the IPCC report and other scientific knowledge. (Australia, interview).

The importance on informants’ knowledge of how the climate crisis has played out locally was demonstrated, for instance, by an Indian activist. He was engaged in the case of wetlands in Tamil Nadu that “retain water when there are floods; they are not wastelands, but they are endangered. […] The 2015 Chennai flood damage could have been minimized if these wetlands were well preserved” (India, interview). The activist thus linked environmental degradation to risks represented by global warming. A German activist had observed “more wildfires […] more droughts, less profitable harvests in the agricultural sector” and mentioned how the heatwaves were affecting the elderly (Germany, interview). In France, an informant pointed out increased levels of droughts, floods, water scarcity and rising sea levels. The Danish activist said that Denmark was “less vulnerable than many other countries” but still reminded that farming could be affected in the future. The Kenyan activist mentioned major droughts*,* which used to occur in a five-year cycle, now with two-year intervals, “and [their] severity has increased”. Furthermore, “drought destroys the biodiversity, “inakausha miti yote” (it dries all the trees), so whenever it rains, water flows and washes everything. It leaves the community vulnerable to floods.” (Kenya, interview).

The activist in Bangladesh referred to a cyclone that devastated large areas of his country when he was seven years old: “The [coastal] area where I live is often hit by storm and cyclones.” Furthermore, his field experience inspired his activism, he said (Bangladesh, interview). The Brazilian activist was preoccupied with fighting a megaproject in an area inhabited by indigenous people, thus demonstrating experiences of especially vulnerable people (Brazil, interview).

### Extreme events, degradation, and anxiety

Activists from the Global South spoke more about extreme events that those from the Global North. They were also more often inspired into action by events and problems in their own countries, consciously or more spontaneously connecting these to the climate science and reports. Connecting the dots, though, may entail more than seeing local extreme events as indicators of climate change. Their answers also spoke of a willingness to engage holistically and globally with the climate crisis and the environment by referring to matters such as species extinction and signs of environment degradation that are not necessarily linked directly to climate change.

Further evidence of the importance of personal experiences were provided by the affective vocabulary employed by many activists. We often heard of an intense interplay of anxiety, stress and even depression, perhaps caused by news consumption and climate crisis predictions. On the other hand, we found expressions of collective and personal enthusiasm provided by the action being taken and the emotive influence of the school strikes. Thus, emotional anxiety often provided them with the impetus to act:” It was like […] sitting on that fear, and anger is not going to do anything” (USA, interview). It was also referred to as a source of authority within the movements: “The strongest voices [in the movement] have a massive emotional attachment to this [activism]” (Denmark, interview). Emotional dynamics also was seen to tie the activists together:” We support each other and feed each other’s anxiety” (Finland, interview).

In the experience of the activists, personal anxiety was also juxtaposed with the thrill of public attention. One intervieweespoke of the sense of seeing “hundreds of thousands of young people, who had different national backgrounds, races, religions, colours” (Indonesia, Interview), “crying as we did the climate scream” (Norway, interview), remembering “our first organisation […] it was very small compared to the other strike, but it was so beautiful” (Turkey, interview), or “leading chants and hearing […] the voices of up to 20,000 people’s strong reply. “(USA, interview).

It is impossible to understand the success of the movement without acknowledging this immediate sense of enpowerment and the feeling among activists that they can do something about their anxiety towards the future. It speaks to the importance of self-reliance and *sense of authenticity* in their messages.

## Contemporary communication landscape: Politics of autonomy

The literature on the political motivation for ubiquitous, digital communication networks of social movements is vast (for a recent review, Caren et al. 2020), but our interviews highlight specific perspectives at least in two ways. First, youth climate activism has emerged in a world where transnational networked communication structures have been an established element of the activists’ life-worlds and shaped their background assumptions about how the world works and what is normal and possible. The idea of a “networked” world and the new roles that this entails (e.g., Castells, 2009: 42–53) is an integral, natural part of the social and political imagination of our respondents’ generation. Second, our respondents are activists who have mobilized specifically around the issue of climate crisis. This thematic aspect of their participation is a crucial part of understanding how they position themselves in the “opportunity structure” and other areas within the field of climate politics.

### Connective action and spectral knowledge

For our respondents, the feeling of being part of a global network of youth activism is an important empowering factor. Direct contacts or organizational negotiations between local actors and spearheading international figures were described as relatively thin and not systematic. Indeed, many of the respondents stressed the autonomy and self-decision-making of local movements: there was often no clear “connection between overseas strikes that are happening” (Australia, interview), and a key characteristic of the movement was “lack of leadership” (Chile, interview). In other words, this meant that “it is necessary to create an original [national] Japanese plan” (Japan, interview) or that “these things would take place even without international movements, without linking with them, attending the joint events etc.” (Slovenia, interview). Of course, the respondents’ emphasis on local initiatives and judgements can partly be a defensive reaction against criticism, as the strikers have been blamed for being driven by fashionable media attention or co-opted by the global movement. Nevertheless, this is also evidence of a de-centralized, “connective” identity towards the global movement: “an organizational principle that is different from notions of collective action based on core assumptions about the role of resources, networks and collective identity” (Bennett & Segerberg, 2013: 53). These de-centralized identities may also be regarded as related to their very different local experiences of climate crisis effects, which inspires locally tailored communication. Furthermore, their desire for local independence could be seen as an element of local, “spectral knowledge”’ that is needed to balance abstract global knowledge production—a critique that has also been directed towards climate science (e.g. Hulme, 2014).

### Negotiating alliances and opponents

The global youth strike movements entered a field of climate politics already crowded with established, powerful actors, organizations, and interests. Cultivating specific relationships with these various parties, in addition to the science, sheds important light on the logic behind the movement.

As one would expect, many global ENGO’s: Greenpeace and 350.org, have provided support to local FFF activists, for instance through workshops to teach activism skills, providing practical support for events or helping to devise media strategies. However, the activists were also careful to sustain a distinction here, arguing that such encounters easily become “a mix of adults and kids; and adults have an implicit authority over children.” (Denmark, interview). They emphasized the egalitarian, mutual exchange of benefits: older activists would offer their experience and younger ones would provide “input about what young people today think” (Finland, interview). There were also references to conflicts,” because some members [of FFF] want to do it only with the students themselves” (Japan, interview). There were even some moments of outright opposition, as from a French respondent: “Greenpeace is 50 years old and there has been no radical change over this time period. Maybe it’s time to adopt a different strategy and to ask for a global and systemic change and not just for incremental measures” (France, interview).

Speaking of their mainstream media relationships, several interviewees underlined how ‘being young’ almost became a separate news value that gave them useful media attention. The downside of this, though, was being treated by journalists as “just kids”’ and not being taken seriously. The *just kids* treatment may be interpreted as a feature of “youngism” (North, 2019, see also Bergmann & Ossewaarde 2020) exercised by media professionals: “[…] we do have formally written and properly articulated demands. I think there’s a bit of still trying to show that it’s a cute child’s movement.” (Canada, interview). Some media would also criticize young people for shirking school.

Overall, the respondents regarded some mainstream media and professional journalists as useful allies who provide public attention and political power for the movement. At the same time, they were highly critical of the ways in which media professionals had covered climate change earlier.

Most activists clearly detached themselves from the established political actors. This defence of their autonomy was often a seen as a balancing act. On the one hand, gestures of public recognition by high-ranking politicians were sometimes presented as highlights of the movement: being mentioned in politicians’ speeches, receiving invitations to delegations, meetings, and networks, and gaining media coverage. “I began to be featured in the media right after the march and felt that I am playing a role in shaping public opinion towards climate change”. (Japan, interview). On the other hand, the activists remain suspicious of this tide of appreciation. Some were annoyed that political parties sometimes joined in on the strikes, prompting a Danish activist to accuse them of an “attempt to hijack our movement” (Denmark, interview) and a Finnish activist described as despicable “politicians [who]…show up for a photo op at strike events, fetching a selfie saying, ‘here we are’; all the while, their deeds are completely incompatible with [our position].” (Finland, interview). Our German respondent said: “Mrs. Merkel herself actually praises what we do but is politically far away from implementing our demands. That is a kind of schizophrenia within these opportunistic forces in the end.” (Germany, interview).

Keeping at a distance also from the more climate-action-friendly political leaders has grown organically from the core logic of the movement. The initial idea of demonstrations at parliaments and not going to school was, after all, directed at the political establishment. On the other hand, the activists also drew attention from the more climate-action-sceptic political camp. Not surprisingly, many youth activists depicted a political landscape whereby right-wing parties, conservatives and populist political players have offered the harshest critiques of their movements. Our Finnish respondent put this ironically, saying some politicians give the impression “that this is some kind of leftist-green conspiracy, and that the stupid kids they should just go back to school as they understand nothing” (Finland, interview). In Norway, “FPU [a right-wing youth party] and the Norwegian oil and gas [lobby] have almost had campaigns against the school strikes.” (Norway, interview).

From these remarks, a synthetic interpretation of how the activists situated themselves into the broader field on climate communication suggests itself. We can see how the social media age and a “connective”, personal attachment logic builds a link between lived experience and public participation and how personal experience and even existential anxiety become cultural, communicative capital that produces and protects a sense of autonomy. This distinct role and the ability to “self-mediate” (Cammaerts, 2018) this identity, in turn, serves as a terrain from which the movement can negotiate its relationship to the other actors that shape its opportunities. The activists have carved out a new, alternative position in the field of politics, distancing themselves both from right-wing populists and the more “climate-policy friendly” politicians.

## Conclusions

Many obstacles to effective climate communication are rooted to the nature and reality of the phenomenon itself as an exceptionally—perhaps uniquely—thorny and complex global problem (e.g. Dryzek et al., 2013; Hulme, 2009). It is a paradigmatic example of a “constitutive” challenge (Jasanoff, 2003) that demands the transformation of many, if not all, sectors and institutions within modern societies, begging us to re-orientate and rethink frames that shape our knowledge, with communication about the world now situated in the balance of time and place (see e.g., Gosh, 2016; Author, 2017; Latour, 2018; Bruhn Jensen, 2018). Still challenges of communicating climate science have troubled academics, including the IPCC itself (Lynn, 2016a, 2016b), and communication professionals for a long time, (e.g. Moser 2009, 2016; Painter 2013; Schäfer & Painter, 2019).

By listening to the activist voices from the recent youth climate movement, we have ventured to understand better both the specific added value of the youth movement to contemporary climate communication and the context that enabled this contribution. Drawing from a global sample of interviews with activists, we have elaborated three elements: 1) the activists’ relationship (and reliance) to climate science (RQ1), 2) the local and personal experiences on which the movement is rooted (despite its global reach) (RQ2), and 3) the opportunities and affordances of the contemporary technical and political communication landscape (RQ3). Each of these elements offers, we think, important insights to climate communication.

A key lesson to climate *science communication* (RQ1), is deceivingly simple: the way the movement actors have positioned themselves as unapologetic ambassadors of the key message of science and its call for urgent climate action. This highlights the value of *consensus as a practical public good*, as a rationally valid, temporally shared understanding and, as a starting point that we should allow to frame the debate about political choices and actions. Stressing the *practical* and political common-sense conclusions and consensus the movement actors have helped to translate scientific knowledge into the realm of public debate. By doing so, they have helped to negotiate the distinction between scientific production of knowledge (and consensus), built on organized uncertainty and doubt, and policy debates that aim towards temporary agreement for action. For our understanding of how better climate science communication works this underlines the importance of actively participatory, and co-productive climate communication where scientific fact and scenarios are translated into meaningful (and powerful) messaging. Of course, this role is not unique and new as such. This is what environmental NGO’s, and climate conscious politicians and their networks have also been involved in for decades. This is what many active scientists have tried to do for a long time (and our respondents reported examples of how the movement inspired some scientists to actively endorse the activists). Thus, to capture more clearly the specific contribution of the youth movement, it is important to look at the nature and identity of the movement itself.

A key feature of the movement (from the perspective of the actors) is its *strong rootedness to concrete, lived experience of the activists* (RQ2). In the interviews, this took several forms. For several respondents, this came through, at the level information and knowledge, in their ability to recognize connections between the main IPCC conclusions and their own local observations that gave global climate science a tangible meaning. More importantly for our argument here, many of them interviewed activists described a strong *affective* dimension in their engagement, showing how activism was often anchored into almost existential, personal, lived experiences of anxiety – and hope. We can argue that the youth movement has played a key role in carving out a space for more public recognition of the fears that dire future predictions must cause also in wider parts of the population. Again, it not alone in articulating climate anxiety, but it has offered a model of translating it to public action. Facing growing uncertainties at a personal level and sharing these publicly will be an important part of public discussion and the ways in which scientific predictions and scenarios of both hope and despair are handled. This psychological aspect of climate debate, on could argue, grows in importance as we begin to realize that we are in some sense “too late”? (Moser, 2020; Bruhn Jensen, 2018). We may view the dynamics between fear and hope (or panic and action) as a practical way of rearticulating the critiques of ‘alarmism’ and dysfunctional ‘disaster’ framing that communication scholars have oftentimes highlighted. In this landscape, the youth movements’ personalized, emotionally loaded and morally strong point is that it is normal to panic, at least sufficiently to spring into action, cannot just be pushed aside as ‘alarmism’, especially when the situation has been established as alarming (Risbey, 2008). Finally, and most importantly, the movement has articulated a new dimension of political representation. They have intensified the climate (science) debate by concretely highlighting politics of *time* and *generations* – and the consequent questions of justice and responsibility. Or put it another way, a simple lesson from the youth movement is to say that they *embody the precautionary principle* of expert assessment. By assuming this role and emphasizing it through striking from *school* (their presupposed path to the future), they have provided a new kind of representation of the future based on climate science and the *urgency* of climate action. Although questions of local experience, personal anxieties and generational justice point to different directions, they can all be seen as resources for the movement to carve out a claim for an *autonomous*, distinct terrain from which an *authentic and distinct voice* could speak with enough authority that it could not be ignored. This voice – previously often ignored (Graham & de Bell, 2021) – and its communicative power is what made and makes the movement also an effective part of communicating climate science and translating it into lessons that bare on meaning structured of everyday life. By assuming the position of living representatives of the future that has been predicted by climate scientists the movement claimed the right and justification to rely on scientific consensus in a way that has been more difficult for other social actors, including the scientists themselves.

Finally, the youth climate movement and its influence has also been *shaped and enabled by its context* (RQ3). The interviews show how the ingredients of autonomy (local knowledge, emotional and temporal claims) seemed to work together. But this was crucially made possible by the contemporary communication infrastructure that supporter “self-mediation” (Cammaerts, 2018) of the movement (both the internal communication between activists and its high public attention). The mix local, personal, generational, emotional and its linkage to hard scientific evidence and consensus is inspiring example of “connective action” in action (Bennett & Segerberg, 2013). However, it is important here to remind that the *core* activities of the movement took place in the street demonstrations, where the activists’ personal feelings of empowerment were often located (this is also an element of the movement that was hit hard by the COVID-19). Understanding the movement through the lens a “relational public” (Starr, 2021), however, also demands that we pay attention to the careful negotiation of alliances and tensions with already existing institutions. This includes recognizing the energizing power of the aggressive dismissal and critique as well as the more supportive reactions of actors that favour climate action, including parts of mainstream media, environmental NGOs, and transnational governance organizations. If we look at the “inherited” landscape of climate politics, perhaps the most important and interesting contribution of the movement does not lie in their confrontations with misogynist populist or networks of climate denial and delay. Rather, the more distinctive and useful element of the movement has been the critique of the capacity of the whole political system (nationally, and internationally) to face the message of climate science and translate that to action.

The youth climate movement articulated an *ethos of a generation.* By connecting youth activists in a loose global network, the movement seems to have at least partly negotiated the pitfalls on being reduced to the global geopolitics of climate change. Science, it seems, played a particular role among these alliances. In a crucial way, the movement supported the communication of the IPCC 1.5 °C report and the global peak of attention that it achieved. In this sense, the movement played a crucial part in science communication of the IPCC—a role that the IPCC perhaps itself could not have played, however skilful or creative it could be in its messaging.

## Annex 1: Contributors to this article, interviewers of youth activists

Australia: David Holmes, Monash University.

Bangladesh: Mofizur Rhaman, Dhaka University.

Brazil: Camila Nobrega, journalist.

Chile: Benjamin Viveros, Universidad Católica de Chile; Katherine Duarte, University of Bergen,

Denmark: Mikkel Eskjaer, Aarhus University.

Finland: Kaarlo Somero, Risto Kunelius, Helsinki University.

France: Elsa Regnier, SciencePo, Paris.

Germany: Torsten Schäfer, Grüner Journalismus.

India: Amutha Kannan, journalist, Chetan Sharma, Datamation Foundation, Delhi.

Indonesia: Oni Sarwono, Universitas Indonesia, Jakarta.

Italy: Susanna Pagiotti, Perugia University.

Japan: Mikhito Tanaka: Waseda University.

Kenya: Joy Kibarabara, Stockholm University.

Norway: Eva Fretheim, journalist, Elisabeth Eide, Oslo Metropolitan University.

Pakistan: Syed M Shakib, FCC University College, Lahore.

Russia: Dmitry Yagodin, Tampere University.

Slovenia: Ilja Tomanic, University of Ljubljana.

South Africa: Alet Janse van Rensburg, journalist/editor.

Sweden: Anna Roosvall, Stockholm University.

Turkey: Elif Ünal, Bianet, Istanbul.

Uganda: Linda Goretti Nassanga, University of Makerere.

USA: Adrienne Russell, University of Washington.

## Footnotes

1 https://thecvf.org/web/climate-vulnerable-forum/ The 48 members come from all parts of the world, except for North America and Europe.

2 The *MediaClimate* network started work in 2008 and has focused both on COP and IPCC coverage (Kunelius et al., 2010; Eide et al., 2012; Kunelius et al., 2017). See website https://mediaclimate.net

3 These interviewees, with the exception of one, belong to countries with current (or past) authoritarian governments, which may be part of the explanation for the local researcher choices, while another part is perhaps these individuals playing an important educative role in building climate literacy (section 4.2). The UN, for statistical purposes, define *youth* as persons between 15 and 24 years of age. See Microsoft Word - YOUTH_Definition_2013-1-23.docx (un.org) Accessed 19.07.2021.

4 In the case of interviewees younger than 18, written consent was acquired from parents. We have quoted all interviewees anonymously.

5 Masson-Delmotte currently heads the IPCC WG I in Assessment Report No 6, presented 09.08.2021.
